# Effects of ensemble and summary displays on interpretations of geospatial uncertainty data

**DOI:** 10.1186/s41235-017-0076-1

**Published:** 2017-10-04

**Authors:** Lace M. Padilla, Ian T. Ruginski, Sarah H. Creem-Regehr

**Affiliations:** 1 University of Utah, Salt Lake City, USA; 2 Department of Psychology, University of Utah, 380 S. 1530 E., Room 502, Salt Lake City, UT 84112 USA

**Keywords:** Ensemble data, Summary display, Visual salience, Hurricane forecast, Visualization cognition, Geospatial data

## Abstract

Ensemble and summary displays are two widely used methods to represent visual-spatial uncertainty; however, there is disagreement about which is the most effective technique to communicate uncertainty to the general public. Visualization scientists create ensemble displays by plotting multiple data points on the same Cartesian coordinate plane. Despite their use in scientific practice, it is more common in public presentations to use visualizations of summary displays, which scientists create by plotting statistical parameters of the ensemble members. While prior work has demonstrated that viewers make different decisions when viewing summary and ensemble displays, it is unclear what components of the displays lead to diverging judgments. This study aims to compare the salience of visual features – or visual elements that attract bottom-up attention – as one possible source of diverging judgments made with ensemble and summary displays in the context of hurricane track forecasts. We report that salient visual features of both ensemble and summary displays influence participant judgment. Specifically, we find that salient features of summary displays of geospatial uncertainty can be misunderstood as displaying size information. Further, salient features of ensemble displays evoke judgments that are indicative of accurate interpretations of the underlying probability distribution of the ensemble data. However, when participants use ensemble displays to make point-based judgments, they may overweight individual ensemble members in their decision-making process. We propose that ensemble displays are a promising alternative to summary displays in a geospatial context but that decisions about visualization methods should be informed by the viewer’s task.

## Significance

Understanding how to interpret uncertainty in data, specifically in weather forecasts, is a problem that affects visualization scientists, policymakers, and the general public. For example, in the case of hurricane forecasts, visualization scientists are tasked with providing policymakers with visual displays that will inform their decision on when to call for mandatory evacuations and how to allocate emergency management resources. In other circumstances, the general public may view hurricane forecasts to make decisions about when and how to evacuate. Even though these types of decisions are costly and have a high impact on health and safety, the literature provides few recommendations to visualization scientists about the most effective way to display uncertainty in hurricane forecasts to a novice audience. Previous research has shown that novice viewers misinterpret widely used methods to visualize uncertainty in hurricane forecasts. The current work examines how novice users interpret two standard methods to display uncertainty in hurricane forecasts, namely ensemble and summary displays. We demonstrate how salient elements of a display – or elements in a visualization that attract attention – can influence interpretations of visualizations. We also provide specific recommendations based on empirical evidence for best practices with each technique.

## Background

Ensemble data is the most commonly used type of forecast data across many scientific domains, including weather prediction and climate modeling (Sanyal et al., 2010). Scientists create ensemble datasets by generating or collecting multiple data values or ‘ensemble members’ (Brodlie, Osorio, & Lopes, 2012; Potter et al., 2009). Then, scientists plot all, or a subset of, the ensemble members on the same Cartesian coordinate plane, creating an ensemble display (Harris, 2000). Despite ensemble display use in scientific practice, it is more common to utilize summary displays for public presentations (Pang, 2008). Scientists construct summary displays by plotting statistical parameters, such as the mean, median, distribution, standard deviations, confidence intervals (CIs) and, with some advanced techniques, outliers, of the ensemble members (Whitaker, Mirzargar, & Kirby, 2013). Among the studies that have attempted to assess the efficacy of ensemble and summary visualizations, there is disagreement about the best method to communicate uncertainty to the general public. This work aims to test the efficacy of both approaches in the context of hurricane forecasts.

Supporters of ensemble displays suggest that there are benefits to this visualization method, including (1) the ability to depict all or the majority of the ensemble data, making a representative portion of the data visually available (Liu et al., 2016); (2) the fact that ensemble displays depict non-normal relationships in the data such as bimodal distributions, perceived as discrete clusters (Szafir, Haroz, Gleicher, & Franconeri, 2016); (3) the preservation of relevant outlier information (Szafir et al., 2016); and (4) the fact that viewers can, in some cases, accurately report some statistical parameters depicted by ensemble displays such as probability distributions (Cox, House, & Lindell, 2013; Leib et al., 2014; Sweeny, Wurnitsch, Gopnik, & Whitney, 2015; Szafir et al., 2016), trends in central tendency (Szafir et al., 2016), and mean size and orientation (Ariely, 2001) (for comprehensive reviews see, Alvarez, 2011; Whitney, Haberman, & Sweeny, 2014). Sweeny et al. (2015) further showed that children as young as four could accurately judge the relative average size of a group of objects. Researchers argue that viewers perceive the aforementioned data parameters in ensemble displays because they can mentally summarize visual features of ensemble displays by perceiving the gist or integrating ensemble data into rich and quickly accessible information (Correll & Heer, 2017; Leib et al., 2014; Oliva & Torralba, 2006; Rousselet, Joubert, & Fabre-Thorpe, 2005). In relation to this, Szafir et al. (2016) detailed four types of tasks (identification, summarization, segmentation, and structure estimation) that are well suited for ensemble displays because they utilize ensemble coding or the mental summarization of data. In line with this work, Correll and Heer (2017) found that participants were effective at estimating the slope, amplitude, and curvature of bivariate data when displayed with scatter plots. In contrast, researchers found that viewers had a strong bias when estimating correlations from scatter plots but also demonstrated that the laws that viewers followed remained similar across variations of encoding techniques and data parameters such as changes in density, aspect ratio, color, and the underlying data distribution (Rensink, 2014, 2016). In sum, there is evidence that adult novice viewers and children can, in some cases, derive statistical information from ensemble displays and that ensemble displays can preserve potentially useful characteristics in the ensemble data.

While previous research indicates that there are various benefits to ensemble displays, there are also some drawbacks. The primary issue with ensemble displays is that visual crowding may occur, which happens when ensemble members are plotted too closely together and cannot be easily differentiated, increasing difficulty in interpretation. While researchers have developed algorithms to reduce visual crowding (e.g., Liu et al., 2016), visual crowding may still occur when all of the ensemble data is plotted.

Summary displays are an alternative to ensemble displays and are suggested to be easier and more effective for users to understand. Work in cartography argues that choropleth maps, which are color encodings of summary statistics such as the average value over a region, are more comprehensible than displaying all of the individual data values (Harrower & Brewer, 2003; Watson, 2013). Michael Dobson argued that the summarization in choropleth maps decreases mental workload and time to perform tasks while improving control of information presentation and pattern recognition (Dobson, 1973, 1980). Beyond choropleth maps, summarization techniques have been developed that can encode advanced summary statistics, such as quartiles, outlier data, and task-relevant features, in ensemble datasets (Mirzargar, Whitaker, & Kirby, 2014; Whitaker et al., 2013).

However, researchers have also documented drawbacks to summarization techniques. First, displays of summary statistics, such as median, mean, and standard deviations, can hide important features in the data such as bimodal or skewed distributions and outliers (Whitaker et al., 2013). Second, summary displays that include boundaries, such as line plots of summary statistics, produce more biased decisions than scatter plots of the same data (Correll & Heer, 2017). Finally, studies have demonstrated that even simple summary displays, such as statistical error bars, are widely misinterpreted by students, the public, and even trained experts (Belia, Fidler, Williams, & Cumming, 2005; Newman & Scholl, 2012; Sanyal, Zhang, Bhattacharya, Amburn, & Moorhead, 2009; Savelli & Joslyn, 2013).

**Fig. 1:**
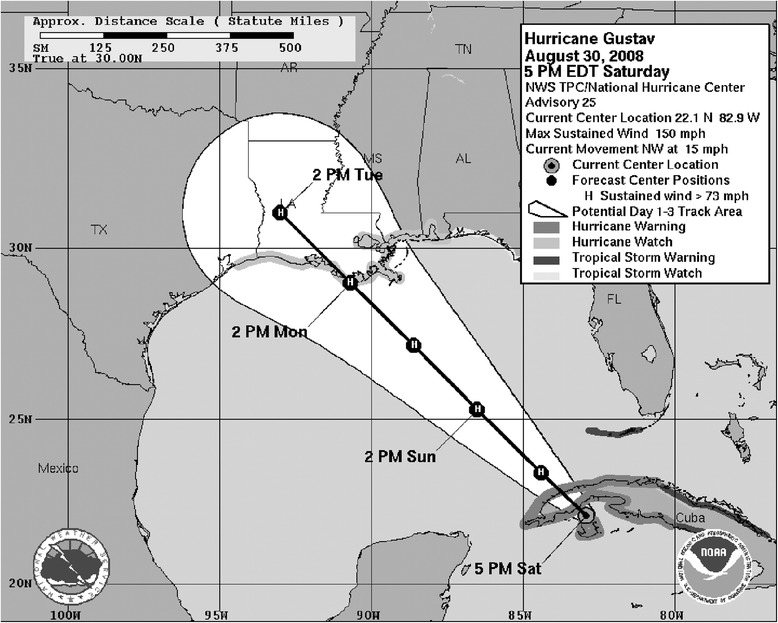
An example of a hurricane forecast cone typically presented to end-users by the National Hurricane Center (http://www.nhc.noaa.gov/aboutcone.shtml)

In the context of hurricane forecasts, there is evidence that summary displays may result in more misinterpretations than ensemble displays (Ruginski et al., 2016). A notable example is the National Hurricane Center’s (NHC) ‘cone of uncertainty’ (Fig. 1).

**Fig. 2:**
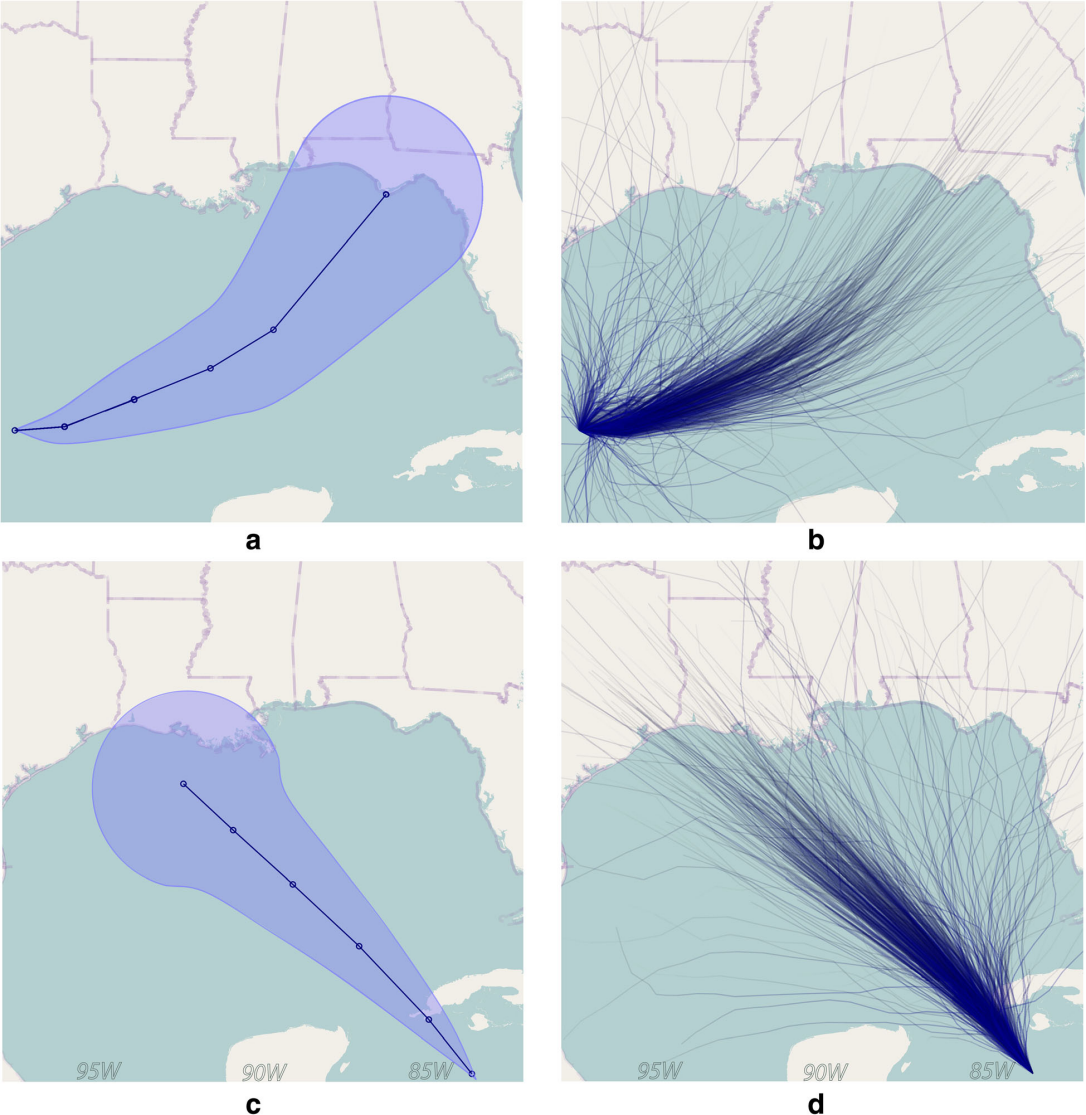
Examples of the cone (**a**, **c**) and ensemble display (**b**, **d**) visualization techniques of hurricane one (**a**, **b**) and two (**c**, **d**)

Forecasters create the cone of uncertainty by averaging a 5-year sample of historical hurricane forecast tracks, resulting in a border where locations inside the boundary have a 66% likelihood of being struck by the center of the storm (Cox et al., 2013). Even though the cone of uncertainty is used by the NHC, it does not follow well-established cartographic principles (e.g., Dent, 1999; Robinson, Morrison, Muehrcke, Kimerling, & Guptill, 1995), including hierarchical organization, which asserts that the level of salience should correspond to the importance of information in a display. However, the cone of uncertainty does support the general view that simplifying complex ensemble data will make decisions easier for users. Ruginski et al. (2016) compared five different encodings of ensemble data (three summary displays, one display of the mean, and one ensemble display) of hurricane forecast tracks, using a task where participants predicted the extent of damage that would occur at a given location. The three summary displays included a standard cone of uncertainty, which had a mean line, a cone without the mean line, and a cone in which the color saturation corresponded to the probability distribution of the ensemble data. Results revealed that, with the summary displays, participants believed that locations at the center of the hurricane that were at a later point in time would receive more damage than at an earlier time point. Strikingly, ensemble displays showed the reverse pattern of responses, with damage rated to be lower at the later time. Further, we found that participants viewing any of the summary displays compared to the ensemble display were significantly more likely to self-report that the display depicted the hurricane growing in size over time. In fact, the cone only depicts a distribution of potential hurricane paths and no information about the size (Cox et al., 2013). One consistency between the three summary displays was the growing diameter of the cone boundaries (as illustrated in Fig. 2a). A possible interpretation of this finding is that viewers focused on the increasing size of the cone, rather than mapping increasing uncertainty to the size of the cone.

More generally, one potential source of the misinterpretation of both summary and ensemble displays is their salient visual features. Salient visual features are defined as the elements in a visualization that attract bottom-up attention (e.g., Itti, Koch, & Niebur, 1998; Rosenholtz & Jin, 2005). Researchers have argued that salience is also influenced by top-down factors (e.g., training or prior knowledge), particularly for tasks that simulate real world decisions (Fabrikant, Hespanha, & Hegarty, 2010; Hegarty, Canham, & Fabrikant, 2010; Henderson, 2007). Hegarty et al. (2010) demonstrated that, in a map-based task, top-down task demands influenced where participants looked on the page, and then salience influenced what information they attended to in the region of interest. This work suggests that both top-down processing and salience guide attention. As described above, a salient visual feature of the cone of uncertainty is the border, which surrounds the cone shape and which grows in diameter with time (Fig. 2a). A salient feature of ensemble displays is the individual ensemble members and their relationship to one another (Fig. 2b). It is possible that the salient features of both the cone of uncertainty and ensemble displays of the same data attract viewers’ attention and bias their decisions (Bonneau et al., 2014).

The motivation for this work was to address both an applied and a theoretical goal. The applied goal was to test whether salient features of summary and ensemble displays contributed to some of the biases reported in prior work (Ruginski et al., 2016), whereas the theoretical goal was to examine whether salient visual features inform how viewers interpret displays. In the case of the cone of uncertainty, viewers may associate the salient increasing diameter of the cone with changes in the physical size of the hurricane. To test this possibility, in the first experiment, we expanded on our previous paradigm by having participants make estimates of the size and intensity of a hurricane with either ensemble or summary displays. In a second experiment, we focused further on the ensemble visualization and judgments of potential damage across the forecast, testing whether the role of the individual lines presented in an ensemble display would be misinterpreted because of their salience in the display. Finally, in a third experiment, we replicate the second experiment and extend the findings beyond a forced choice task.

## Experiment 1

In line with our prior work (Ruginski et al., 2016), we hypothesized that participants viewing the cone of uncertainty would report that the hurricane was larger at a future time point. It was an open question whether judgments of intensity would also be associated with the depicted size of the cone. We predicted that those viewing the ensemble display would report that the size and intensity of the storm remained the same in the future because the size cue from the cone was not present. On the other hand, for ensemble hurricane track displays (Fig. 2b, d), it is possible that the individual tracks and their relationship to one another are the salient features used to interpret the hurricane forecast. The tracks in the ensemble display employed by Ruginski et al. (2016) became increasingly farther apart as the distance from the center of the storm increased, which could be associated with a decrease in perceived intensity of the storm. We predicted that participants viewing the ensemble display would believe that the storm was less intense where the individual tracks were farther apart (an effect of distance from the center of the storm). However, because the cone of uncertainty lacks this salient spread of tracks, we predicted that judgments of intensity when viewing the cone would not be affected by distance from the center of the storm.

**Fig. 3:**
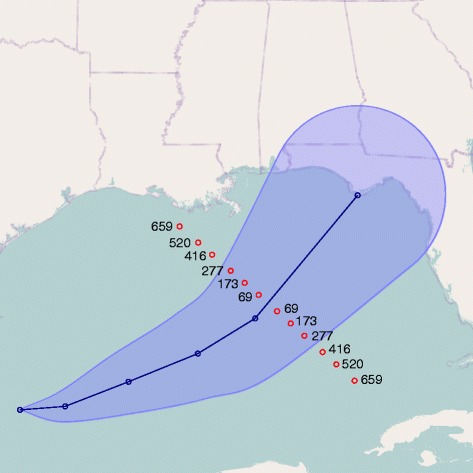
An example of the cone visualization, shown with the 12 possible oil rig locations. Only one location was presented on each trial (and km were not presented)

### Methods

#### Participants

Participants were 182 undergraduate students currently attending the University of Utah who completed the study for course credit. Three individuals were excluded from final analyses for failing to follow instructions. Of the 179 included in analyses, 83 were male and 183 were female, with a mean age of 21.78 years (SD = 5.72). Each participant completed only one condition, either size task with cone (n = 40), size task with ensemble display (n = 42), intensity task with cone (n = 48), or intensity task with ensemble display (n = 48).

#### Stimuli

Stimuli were presented online using the Qualtrics web application (Qualtrics [Computer software], 2005). In each trial, participants were presented with a display depicting a hurricane forecast. The hurricane forecast images were generated using prediction advisory data from two historical hurricanes, available on the NHC website (http://www.nhc.noaa.gov/archive). The cone of uncertainty and an ensemble display technique were both used to depict the two hurricanes (Fig. 2).

A custom computer code was written to construct the summary and ensemble displays, using the algorithm described on the NHC website (http://www.nhc.noaa.gov/aboutcone.shtml). The ensemble and summary displays were created using the code of Cox et al. (2013). The resulting displays were a subset of the five visualization techniques used in Ruginski et al. (2016), which depicted two hurricanes and were randomly presented to participants. All were digitally composited over a map of the U.S. Gulf Coast that had been edited to minimize distracting labeling. These images were displayed to the subjects at a pixel resolution of 740 × 550. A single location of an ‘oil rig’ depicted as a red dot was superimposed on the image at one of 12 locations defined relative to the centerline of the cone and the cone boundaries. We chose the following distances to place the oil rigs relative to the centerline of the cone, 69, 173, 277, 416, 520, and 659 km (Fig. 3), which correspond to 0.386, 0.97, 1.56, 2.35, 2.94, and 3.72 cm from the center line of the hurricane on the map.

**Fig. 4:**
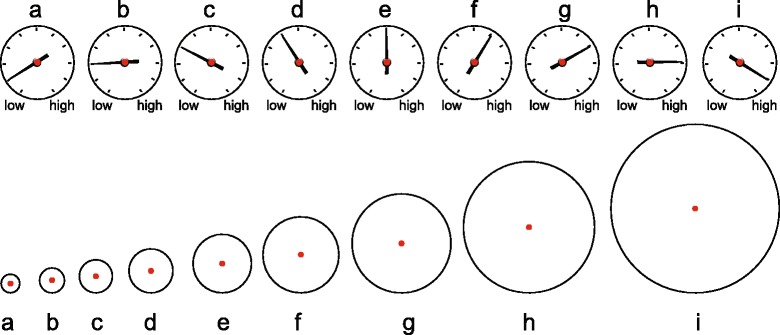
An example of the visual depiction of the Likert scales, which depicts intensity with gauges (top) and size with the diameter of the circle (bottom)

Relative points with respect to the center and cone boundary were chosen so that three points fell outside the cone boundary (277, 173, and 69 km), three points fell within the cone boundary (416, 520, and 659 km), and so that no points appeared to touch the visible center line or boundary lines. Underneath the forecast, a scale ranging from A to I was displayed along with visual depictions. For the intensity task, the scale was indicated by gauges, and for the size task the scale was indicated by circles (Fig. 4). Each circle was scaled by 30% from the prior circle. Each gauge was scaled by 1 ‘tick’ from the prior gauge. The starting size and intensity of the hurricane were overlaid on the beginning of the hurricane track forecast for each trial. Three starting sizes and intensities (C, E, G) were presented in a randomized order.

#### Salience assessment

**Fig. 5:**
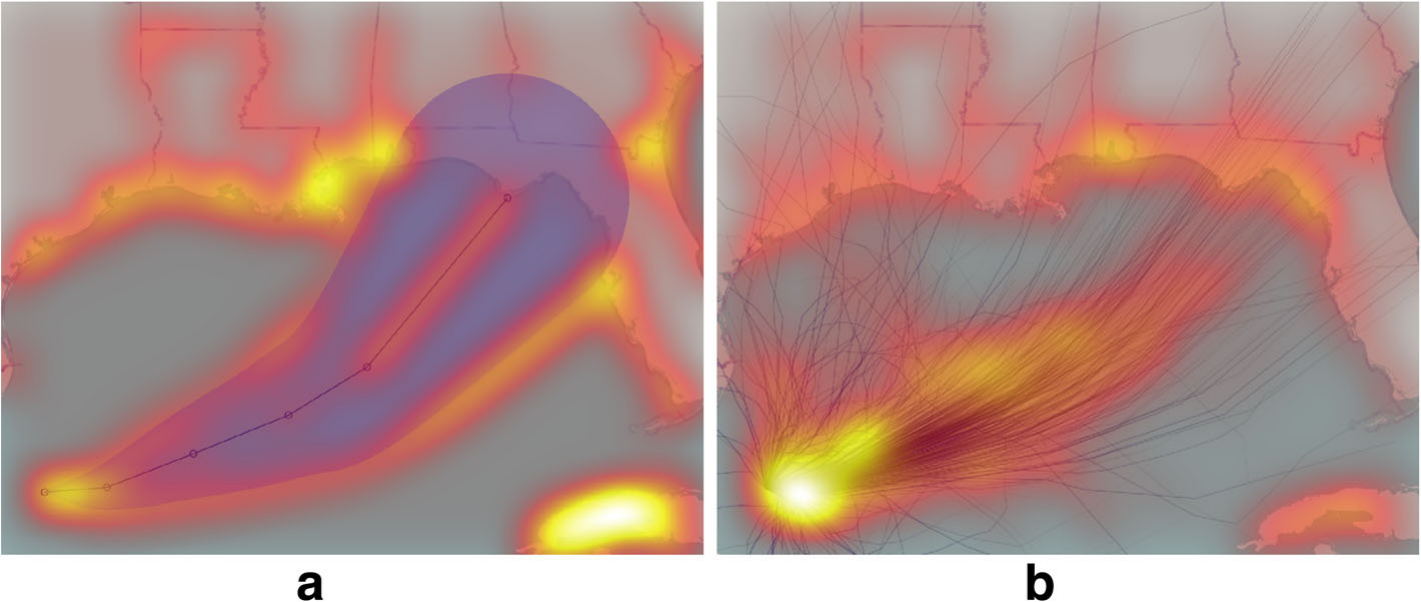
Example of the visual output generated using the Itti et al. (1998) salience model, which shows example stimuli used in this experiment. Brighter coloration indicates increased salience. **a** The summary display. **b** The ensemble display

To test the previously stated prediction about salience of features of ensemble and summary displays, we utilized the Itti et al. (1998) salience model. Prior research has employed the Itti et al. (1998) salience model to test the salience of cartographic images and found that this model is a reasonable approximation of bottom-up attention (Fabrikant et al., 2010; Hegarty et al., 2010). The Itti et al. (1998) salience model was run in Matlab (2016, Version 9.1.0.441655) using the code provided by Harel et al., (2007). The results of this analysis suggest that the most salient visual features of the cone of uncertainty are the borders of the cone and the centerline (Fig. 5a). Additionally, the salient visual features of the ensemble display are the relative spread of hurricane tracks (Fig. 5b).

#### Design

We utilized a 2 (visualization type) × 2 (hurricane) × 3 (starting size or intensity) × 12 (oil rig location) mixed factorial design for each task (size and intensity). Hurricane starting size or intensity and the oil rig location were within-participant variables, resulting in a total of 72 trials per participant. Participants were randomly assigned to one of two visualization conditions (summary or ensemble display) and one of two tasks (size or intensity) as between-participant factors.

#### Procedure

Individuals were first given a simple explanation of the task and visualization. Participants completing the size task were provided with the following instructions:

“*Throughout the study you will be presented with an image that represents a hurricane forecast, similar to the image shown above. You will be provided with the initial hurricane size (diameter) at a particular point in time, indicated by the circle shown at the apex (beginning) of the hurricane forecast. An oil rig is located at the red dot. Assume that the hurricane were to hit the oil rig (at the red dot). Your task will be to select the size that best represents what the hurricane’s diameter would be when it reaches the location of the oil rig.*”

Additionally, each trial included the text as a reminder of the task, “*Assume that the hurricane were to hit the oil rig (at the red dot). Your task is to select the size that best represents what the hurricane’s diameter would be when it reaches the location of the oil rig.*”

For the intensity task, participants were provided the instructions:

“*Throughout the study you will be presented with an image that represents a hurricane forecast, similar to the image shown above. You will be provided with the initial hurricane wind speed at a particular point in time, indicated by the gauge shown at the apex (beginning) of the hurricane forecast. As the arm of the gauge rotates clockwise, the wind speed increases. For example, gauge A represents the lowest wind speed and gauge I the highest wind speed. An oil rig is located at the red dot. Assume that the hurricane were to hit the oil rig (at the red dot). Your task will be to select the gauge that best represents what the hurricane’s wind speed would be when it reaches the location of the oil rig.*”

Each trial also contained the instructions, “*Assume that the hurricane were to hit the oil rig (at the red dot). Your task is to select the gauge that best represents what the hurricane’s wind speed would be when it reaches the location of the oil rig.*”

Following the instructions, participants completed all of the trials presented in a different random order for each participant. Finally, participants answered questions related to comprehension of the hurricane forecasts. These included two questions specifically relevant to the current research question: “*The display shows the hurricane getting larger over time*.” and “*The display indicates that the forecasters are less certain about the path of the hurricane as time passes*.” These questions also included a measure of the participants’ understanding of the response glyphs used in the experiment by asking them to indicate which of two wind gauges had a higher speed or to match the size of circles. Participants who did not adequately answer these questions were excluded from the analysis (two participants for the wind speed gauges, one for the size circles).

#### Data analysis

Multilevel models (MLM) were fit to the data using Hierarchical Linear Modeling 7.0 software and restricted maximum likelihood estimation procedures (Raudenbush & Bryk, 2002). Multilevel modeling is a generalized form of linear regression used to analyze variance in experimental outcomes predicted by both individual (within-participants) and group (between-participants) variables. A MLM was appropriate for modeling our data and testing our hypotheses for two major reasons. Firstly, MLM allows for the inclusion of interactions between continuous variables (in our case, distance) and categorical predictors (in our case, the type of visualization). Secondly, MLM uses robust estimation procedures appropriate for partitioning variance and error structures in mixed and nested designs (repeated measures nested within individuals in this case).

We transformed the dependent variable before analysis by calculating the difference between the starting value of the hurricane (either size or intensity) and the participant’s judgment. A positive value of the difference score represents an increase in judged size or intensity. In addition, although an ordinal variable by definition, we treated the dependent variable Likert scale as continuous in the model because it contained over five response categories (Bauer & Sterba, 2011).

For the distance variable, we analyzed the absolute value of oil rig distances, regardless of which side of the hurricane forecast they were on, as none of our hypotheses related to whether oil rigs were located on a particular side. We divided the distance by 10 before analysis so that the estimated model coefficient would correspond to a 10-km change (rather than a 1-km change). The mixed two-level regression models tested whether the effect of distance from the center of forecasts (level 1) varied as a function of visualization (level 2). Visualization was dummy coded such that the cone visualization was coded as 0 and the ensemble display as 1. We tested separate models for the intensity and size tasks. Self-report measures of experience with hurricanes and hurricane prone regions were also collected. As the participants were students at the University of Utah, so few had experienced a hurricane (3%) or had lived in hurricane-affected regions (7%) that we did not include these measures as covariates.

### Results – Size

Level 1 of our multilevel model is described by:

$$ Chang{e}_{ij}={\beta}_{0j}+{\beta_{1j}}^{\ast}\left( Distanc{e}_{ij}\right)+{r}_{ij}; $$

and level 2 by:

$$ {\upbeta}_{0\mathrm{j}}={\upgamma}_{00}+{\upgamma_{01}}^{\ast}\left({\mathrm{Visualization}}_{\mathrm{j}}\right)+{\mathrm{u}}_{0\mathrm{j}} $$

$$ {\upbeta}_{1\mathrm{j}}={\upgamma}_{10}+{\upgamma_{11}}^{\ast}\left({\mathrm{Visualization}}_{\mathrm{j}}\right)+{\mathrm{u}}_{1\mathrm{j}} $$

Where i represents trials, j represents individuals, and the β and γ are the regression coefficients. The error term r_ij_ indicates the variance in the outcome variable on a per trial basis, and u_0j_ on a per person basis. Though people are assumed to differ on average (u_0j_) in the outcome variable, we tested to determine whether the effect of distance differed per person (u_1j_) using a variance-covariance components test. We found that the model including a random effect of distance fit the data better than the model not including this effect, and so the current results reflect that model (χ^2^ = 955.95, *df* = 2, *P* < 0.001). Including this term allowed us to differentiate between the variance accounted for in judgments specific to a fixed effect of distance and the variance accounted for in judgments specific to a random effect of person.

**Fig. 6:**
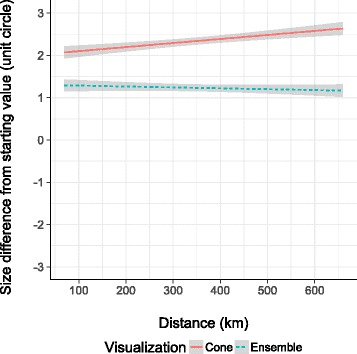
The effect of distance from center and visualization type on change in size judgments. Grey shading indicates ± 1 standard error. Accurate interpretation would be indicated by a ‘0’ change score. A one-unit change represents a one-step change in circle size along a 9-point scale (see Fig. 4 for the 9-point scale)

Our primary hypothesis was that we would see greater size judgments with the cone compared to the ensemble display, reflecting a misinterpretation that the hurricane grows over time. Consistent with this prediction, we found a significant main effect of visualization type on average change in size judgments (*γ*_*01*_ = −0.69, standard error (SE) = 0.33, *t-ratio* = −2.08, *df* = 80, *P* = 0.04). This effect indicates that, at the center of the hurricane, individuals viewing the cone visualization had a 0.69 greater increase in their original size judgment compared with individuals viewing the ensemble visualization (Fig. 6). However, the oil rig distance from the center of the storm did not significantly alter change in size judgments (*γ*_*10*_ = 0.01, SE = 0.01, *t-ratio* = 1.43, *df* = 80, *P* = 0.16) and the effect of distance from the center of the storm on change in size judgments did not differ based on visualization type (*γ*_*11*_ = −0.01, SE = 0.01, *t-ratio* = −1.32, *df* = 80, *P* = 0.19). Further, the main effect of visualization type on the average change in size judgment was also supported by results of the post-test question. A *t-*test, in which yes was coded as 1 and no as 0, revealed that participants viewing the cone (*M* = 0.70, SE = 0.04) were significantly more likely to report that the display showed the hurricane getting larger over time compared to the ensemble display (*M* = 0.39, SE = 0.05), *t*(176) = 4.436, *P* < 0.001, 95% CI 0.17–0.45, Cohen’s *d =* 0.66.

### Results – Intensity

The multilevel model used for the intensity data included the exact same variables as the size model. Similar to the first model, we found that the model including a random effect of distance fit the data better than the model not including this effect, and so the current results reflect that model (χ^2^ = 704.81, *df* = 2, *P* < 0.001).

**Fig. 7:**
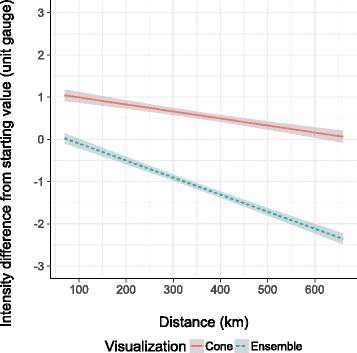
Simple slopes of the interaction between distance and visualization type on change in intensity judgments. Grey shading indicates ± 1 standard error. Accurate interpretation would be indicated by a ‘0’ change score. A one-unit change represents a one-step change in gauge intensity along a 9-point scale (see Fig. 4 for the 9-point scale)

For intensity, we expected to see a greater effect of distance from the center of the storm on judgments with the ensemble display compared to the cone, reflecting participants’ attention to the increasing spread of tracks as the distance from the center increase for the ensemble display. First, we found a significant main effect of visualization type on average change in intensity judgments (*γ*_*01*_ = −0.85, SE = 0.33, *t-ratio* = −2.58, *df* = 95, *P* = 0.01). This indicates that, at the center of the hurricane, individuals viewing the cone visualization increased their intensity judgment by 0.85 (almost a full wind gauge) more than those who viewed the ensemble visualization at the center of the hurricane. Second, we found a significant main effect of distance from the center of the storm (*γ*_*10*_ = −0.02, SE = 0.01, *t-ratio* = −3.28, *df* = 95, *P* = 0.001), which is qualified by a significant cross-level interaction between distance and visualization type (*γ*_*11*_ = −0.02, SE = 0.01, *t-ratio* = −3.33 *df* = 95, *P* = 0.001). To decompose the interaction between distance from the center of the storm and visualization type, we computed simple slope tests for the cone and ensemble visualizations (Fig. 7). This revealed that the association between distance from the center of the hurricane and change in intensity judgment is different from zero for each visualization (cone visualization: Estimate = −0.02, SE = 0.01, χ^2^ = 64.74, *P* < 0.001; ensemble visualization: Estimate = −0.04, SE = 0.004, χ^2^ = 10.74, *P* = 0.001) and stronger for the ensemble visualization (χ^2^ = 101.89, *P* < 0.001). This result suggests that judgments of intensity decreased with distance more for the ensemble display than for the cone, consistent with a focus on the relative spread of hurricane tracks. In addition, using a *t-*test, a post-test question revealed that participants viewing the ensemble display (*M* = 0.53, SE = 0.04) were more likely to report that the display indicated the forecasters were less certain about the path of the hurricane over time compared to the cone (*M* = 0.39, SE = 0.05), *t*(176) = −1.97, *P* = 0.04, 95% CI −0.29 to −0.0003, Cohen’s *d* = 0.29.

### Discussion

The results of this experiment showed that novice users interpret the size and intensity of a hurricane represented by ensemble and summary displays differently. Our prior work showed different damage ratings over time with the cone compared to the ensemble display, but it was unclear whether these were being driven by interpretations of size or intensity because a more general concept of ‘damage’ was used (Ruginski et al., 2016). In the current study, we found a similar pattern of greater increase in both size and intensity reported at the center of the hurricane with the cone, compared to the ensemble display. Furthermore, we found an effect of decreasing intensity judgments with distance from the center of the storm that was greater for the ensemble display than for the cone.

These findings support our hypothesis that a salient feature of the cone is the border that shows the diameter of the cone, which is more likely to influence viewers’ beliefs that the storm is growing over time compared to the ensemble display, which does not have this visually salient feature. We saw evidence of the participants’ beliefs that the cone represented the storm growing in size with both objective judgments of size (which increased more relative to judgments made using the ensemble display) and self-reported interpretations of the cone of uncertainty. Our second hypothesis that participants viewing the ensemble display would believe that the storm was less intense where the individual tracks were farther apart was supported by results of the intensity task conditions. Here, while intensity ratings were higher for the cone compared to the ensemble display, the rate of decrease in ratings of intensity as distance from the center of the storm increased was greater for the ensemble display than the cone. Together, these findings demonstrate that, in the context of hurricane forecasts, the salient visual features of the display bias viewers’ interpretations of the ensemble hurricane tracks.

More generally, we suggest that summary displays will be most effective for cases in which spatial boundaries of variables such as uncertainty cannot be misconstrued as presenting physical boundaries. In contexts like cartography, where spatial layouts inherently represent physical space, ensemble displays provide a promising alternative to summary displays. Although our findings suggest that ensemble displays seem to have some advantages over summary displays to communicate data with uncertainty in a geospatial context, it may also be the case that ensemble displays provoke additional unintended biases. We tested one potential ensemble display bias in Experiment 2.

## Experiment 2

While the findings of Experiment 1 suggested that viewers of the ensemble visualization are less likely to believe that the hurricane is growing in size, it is possible that ensemble displays also elicit unique biases. One possible bias is that the individual tracks of an ensemble display can lead a viewer to overestimate the impact of the hurricane for locations covered by a path. The storm tracks presented are only a sampling of possible ways the hurricane could go and not an exhaustive list of all routes. It would be a misconception to believe that a hurricane would travel the full extent of any one track. Further, it would also be incorrect to believe that locations that are not covered by a path have little to no possibility of being hit by the storm. Rather, the relative density of tracks indicates the comparative probability of a hurricane being in a given region at future time points.

**Fig. 8:**
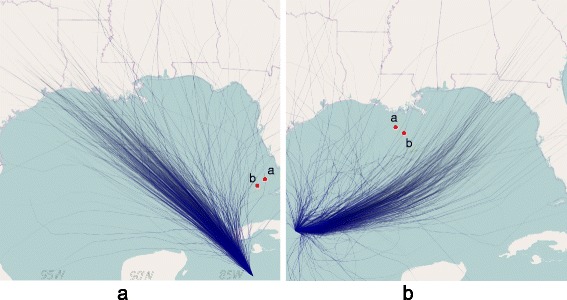
Examples of the stimuli used in Experiment 2 depicting two hurricanes. **a** Condition in which the farther rig from the center of the storm was located on a hurricane track. **b** Condition where the closest rig was located on a hurricane track

To test whether viewers’ decisions are biased by the individual paths of the ensemble visualization, we conducted a second experiment in which the locations of the oil rigs were changed so that one oil rig was always superimposed on a hurricane path. We examined whether viewers would maintain the strategy to rate higher damage closer to the center of the storm, as reported in Ruginski et al. (2016) (i.e., selecting the closest rig to the center), or whether the salience of the ensemble track location would decrease the strength of the distance-based strategy (i.e., selecting the rig that was superimposed on a hurricane path, even when located farther away from the center of the storm). In this experiment, participants were presented with two oil rigs, one that was located on a hurricane path and one that was either closer (Fig. 8a) or farther from the center of the storm (Fig. 8b) than the one that was located on the path.

Participants were then asked to decide which of the two oil rigs would receive the most damage. Our hypothesis was that the likelihood of choosing the rig closer to the center of the storm would decrease if the rig farther from the center of the storm fell on a hurricane path, supporting the notion that the individual paths are salient features of the ensemble display that could lead to biased responses. In the rest of the paper we will refer to the close oil rig, meaning the oil rig that is closer to the center of the hurricane forecast display, and the farther oil rig, which is the rig farther away from the center of the hurricane forecast than the closer oil rig.

### Methods

#### Participants

Participants were 43 undergraduate students currently attending the University of Utah who completed the study for course credit; 12 participants were male and 31 were female, with a mean age of 23.56 years (SD = 7.43).

#### Stimuli

Stimuli were presented using the previously detailed approach. On each trial, participants were presented with a display depicting a hurricane forecast and two oil rigs (Fig. 8). The distance between the oil rigs was roughly 100 km and remained constant for all of the trials. The 16 locations of the rig pairs were chosen selectively in areas where one rig was always located on a track and the other oil rig was at the same time point but not on a track, with an equal number of locations on each side of the hurricane. The rig on the track was either closer to the center or farther from the center relative to the rig that was not touching a track. Underneath the forecast, radio buttons were presented that allowed participants to indicate which oil rig they believed would receive the most damage. Damage was used for the response measure because it was found that participants were more likely to use a strategy based on distance from the center of the hurricane when making judgments about damage. This measure allowed us to determine if the colocation of an oil rig and a hurricane track modified the types of distance-based damage judgments reported in Ruginski et al. (2016).

#### Design

We utilized a within-subjects design, 2 (close oil rig on line or far oil rig on line) × 2 (hurricane) × 16 (oil rig pair locations), resulting in a total of 32 trials per participant. Oil rig on line refers to whether the closer or farther oil rig from the center of the hurricane were located on the hurricane track.

#### Procedure

Individuals were first given a simple explanation of the task and visualization.

“*Throughout the study you will be presented with an image that represents a hurricane forecast, similar to the image shown above. An oil rig is located at each of the two red dots. Your task is to decide which oil rig will receive more damage based on the depicted forecast of the hurricane path.*”

Additionally, each trial included the text, “*Your task is to decide which oil rig will receive the most damage from the hurricane*.” Following the instructions, participants checked a box indicating which oil rig they believed would receive the most damage. The trials were presented in a different random order for each participant. Finally, participants answered demographic questions and questions related to hurricane experience.

#### Data analysis

A multilevel logistic regression model was fit to the data using the lme4 package in R and maximum likelihood Laplace approximation estimation procedures (Bates, Maechler, Bolker, & Walker, 2015). A logistic MLM was appropriate for modeling our data and testing our hypotheses because it uses robust estimation procedures appropriate for partitioning variance and error structures in mixed and nested designs (repeated measures nested within individuals in this case) for binary outcomes (choosing which oil rig would receive more damage in this case).

Level 1 of our multilevel model is described by:

$$ Close\kern0.5em Strateg{y}_{ij}={\beta}_{0j}+{\beta_{1j}}^{\ast}\left( Far\  Rig\  On\  Line\right)+{r}_{ij}; $$

and level 2 by:

$$ {\upbeta}_{0\mathrm{j}}={\upgamma}_{00}+{\mathrm{u}}_{0\mathrm{j}} $$

$$ {\upbeta}_{1\mathrm{j}}={\upgamma}_{10} $$

*Far Rig On Line* was dummy coded such that the farther rig overlapping with a line corresponded to 1, while the closer rig being on the line corresponded to 0. Our outcome variable, *Close Strategy*, was coded such that selecting the close oil rig to receive more damage corresponded to 1 and selecting the far oil rig to receive more damage corresponded to 0. We found that the model not including a random effect of On Line fit the data better than the model including this effect, and so the current results reflect the former (χ^2^ = 5.79, *df* = 1, *P* = 0.02). This indicates that there was a consistent fixed effect of On Line across people.

The participants had very high odds of deciding that the closer oil rig would receive the most damage when the closer oil rig was on the line (and by design, the farther oil rig was not on a line) (*γ*_*00*_ = 5.75, SE = 0.52, odds ratio (OR) = 314.19,^1^ z = 11.19, *P* < 0.001). Expressed in terms of predicted probability, this effect indicates that participants chose the closer oil rig to receive more damage 99.68% of the time when the closer oil rig was on a line (Fig. 8). This very high proportion makes sense, as this condition combined properties of close location to the center and a location falling on the path. Importantly, our model indicated a strong effect of Far Rig On Line, such that predicted probability of choosing the closer oil rig as receiving the most damage decreased to 64.15% when the farther oil rig was on the line (*γ*_*10*_ = −5.17, SE = 0.37, OR = 0.006, z = −13.85, *P* < 0.001; Fig. 8). In this condition, the far oil rig was chosen in 304 of the 688 trials, compared to only 12 of the 688 trials when it was not on the line.

**Fig. 9:**
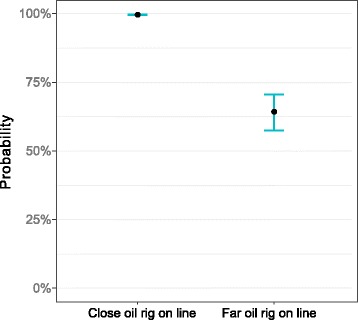
Predicted probabilities of choosing the close oil rig to receive more damage. Bars represent 95% confidence intervals. Accurate interpretation would be to choose the close oil rig 100% of the time

In other words, while participants chose the closer oil rig more often in both conditions, the result that the tendency to choose the farther rig increased by about 35% when the farther rig fell on a visual path strongly supports the use of the individual path as a salient feature influencing decisions (Fig. 9).

### Discussion

We found that non-experts almost always chose the closer oil rig to the center of the hurricane forecast when the oil rig fell on an individual hurricane track, consistent with prior work showing a strategy to report more damage to locations close to the center (Ruginski et al., 2016). However, when the farther oil rig visually overlapped with a single ensemble track, judgments were significantly biased by the individual path, decreasing the likelihood of choosing the close location. The results of the second study suggest that ensemble displays have their own set of interpretation biases, as individual ensemble members can be over weighted in participants’ judgments.

## Experiment 3

In an effort to replicate the prior study and test if the findings were robust to a non-forced choice task, a third study was conducted that was identical to Experiment 2 but with an additional response option of ‘Equal Damage’. By adding an ‘Equal Damage’ response participants could indicate that neither oil rig A or B would receive more damage. The same methods and data analysis were used as Experiment 2. Participants were 35 undergraduate students currently attending the University of Utah who completed the study for course credit; 10 participants were male, and 24 were female, with a mean age of 22.06 years (*SD* = 4.5).

### Results

As in Experiment 2, we used a multilevel logistic regression model to determine the impact of the colocation of an ensemble track and an oil rig. Prior to analysis, trials for which participants reported ‘Equal Damage’ (219 trials, 19.55% of total) were removed. Of the trials where participants reported equal damage, 79 occurred when the close rig was on a line and 140 occurred when the far rig was on a line. Models including fixed effects only and random effects fit the data equally well and results detail the more parsimonious model not including the random effect (χ^2^ = 0, *df* = 1, *P* = 1.00). This indicates that there was a consistent fixed effect of the oil rig touching an ensemble track across people.

**Fig. 10:**
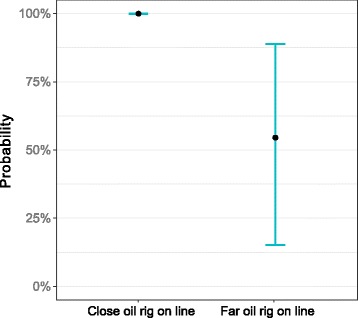
Predicted probabilities of choosing the close oil rig to receive more damage. Bars represent 95% confidence intervals. Accurate interpretation would be to choose the close oil rig 100% of the time

Consistent with Experiment 2, participants had high odds of deciding that the closer oil rig would receive the most damage when it was on the line (*γ*_*00*_ = 10.94, SE = 1.52, OR = 56387.34,^2^ z = 7.2, *P* < 0.001). In other words, participants indicated that the closer oil rig would receive more damage 99.99% of the time when it was on a line. This finding replicates the results of our prior experiment. Further, our results showed a similar effect compared to Experiment 2 for Far Rig On Line, such that predicted probability of choosing the closer oil rig as receiving the most damage decreased to 54.59% when the farther oil rig was on the line (*γ*_*10*_ = −10.76, SE = 1.29, OR = 0.00002, z = −8.36, *P* < 0.001; Fig. 10). In this condition, the far oil rig was chosen in 238 of 420 trials, compared to only 1 of 481 trials when it was not on the line. In sum, Experiment 3 replicates the take home points of Experiment 2, but the SE increased in Experiment 3. It is likely that including the response option of ‘Equal Damage’ increased the variability of the responses by decreasing sample size (reducing trials) and choosing the far rig more often (almost 50–50) for those trials that were not decided as equal damage.

### Discussion

In Experiment 3, we replicated Experiment 2, showing that participants were significantly biased by the colocation of an oil rig and an individual ensemble track. In the third study, in 19.55% of trials, individuals believed that the two oil rigs would receive equal damage, and about twice as many of these trials occurred when the far oil rig was on the line, providing additional evidence that the line competes with proximity to the center in evaluation of damage. For the rest of the trials where individuals chose either the close or far oil rig, results were consistent with Experiment 2, showing a decrease in the likelihood of choosing the close location when the far oil rig fell on the line. Together, these studies demonstrate that decisions about ensemble displays of hurricane forecast tracks change when making judgments about specific points that intersect with a track. More broadly, this work suggests that individual members of an ensemble display may be overweighed when the ensemble member happens to overlap with a point of interest. For example, individuals may be more likely to evacuate or take precautionary actions if a hurricane forecast track overlaps with their own town, but feel less concerned if not. These results suggest that visualization scientists should consider using ensemble displays in cases where users do not need to make decisions about specific points that may be influenced by an ensemble member. Instead, ensemble displays may be best suited for cases in which viewers are making judgments about patterns in the data or about areas, which is consistent with tasks proposed for ensemble displays by Szafir et al. (2016).

Our findings may be influenced by the nature of the task in a geospatial context, where asking about a single point biases users towards more of an outlier-identification strategy (Szafir et al., 2016). Future work involving interpretation of geospatial uncertainty may help to disentangle this by implementing tasks that require individuals to make judgments about larger areas of space (such as a county), which may force individuals to summarize the visualization and be less biased by individual tracks. Correll and Heer (2017) provide support for the claim that tasks influence the nature of biases by demonstrating that viewers are not affected by outliers when making judgments about the overall trends in ensemble data.

## General discussion

Our first study demonstrated that novice users interpret the size and intensity of a hurricane represented by an ensemble display and the cone of uncertainty differently, with relative lower size and intensity judgments over time for the ensemble display compared to the cone. These findings support our hypothesis that viewers of the cone of uncertainty are more likely to incorrectly believe that the visualization depicts the hurricane growing over time, consistent with the results of Ruginski et al. (2016). Furthermore, in the intensity task condition, we found a stronger effect of distance from the center of the hurricane for the ensemble display than for the cone. This result is in line with our predictions, providing evidence that a salient feature of the ensemble display is the tracks and their relationship to one another. In sum, these studies suggest that the type of visualization technique used to depict hurricane tracks significantly influences viewers’ judgments of size and intensity – these effects are likely driven by the salient features of the displays, consistent with prior work (Correll & Heer, 2017; Newman & Scholl, 2012). Beyond hurricane forecasts, this work proposes that salient visual features in a display can attract viewers’ attention and bias their decisions. Attention may bias viewers’ judgments by manipulating the relative importance of features. Viewers may overweight the importance of salient features because they are attending to them more or they may devalue other features that they pay less attention to.

Despite their benefits, ensemble displays are not free of biases that negatively affect uncertainty comprehension. Our second and third studies found that, while novice users predominantly make judgments as if ensemble displays are distributions of probable outcomes, they also indicate that locations that are touching an individual ensemble track will receive more damage. However, we speculate that individual ensemble members may only influence judgments of specific points and may not influence users making judgments about areas. This assertion is consistent with work that suggests ensemble displays are well suited to conveying the gist of a scene (Correll & Heer, 2017; Oliva & Torralba, 2006; Rousselet et al., 2005). Further, the types of tasks that Szafir et al. (2016) propose for ensemble displays all include identifying patterns in groups of data that are spatially organized rather than point-based judgments. This suggests that visualization scientists should consider the types of tasks that their users will be completing when selecting the appropriate visualization technique, and that ensemble displays are most appropriate for tasks that do not require judgments about specific points.

Understanding human reasoning with static ensemble displays is a necessary first step to unpacking ensemble cognition; however, many visualization scientists may desire to present ensemble displays as animations or time-varying displays (Liu et al., 2016). Time-varying displays continually update the visualization with simulations, fading simulations out as a function of their time on the screen, which could reduce the salience of individual simulations. Directly manipulating the salience of features with animations, in line with Fabrikant et al. (2010) and Hegarty et al. (2010), is a possible future direction for this work. While animations may reduce biases produced by individual tracks, they may not be entirely beneficial (Tversky, Morrison, & Betrancourt, 2002) and often show little benefit when learning information from visualizations (Hegarty, Kriz, & Cate, 2003). However, the aforementioned work predominantly examined process diagrams and the negative impact of animations may not generalize to decision-making with uncertainty visualizations. Additionally, many animated visualization techniques also include user interaction capabilities. To determine the specific contributions of animation and user interaction to ensemble cognition, a systematic study is needed that tests both area and point-based judgments using these techniques.

Future work is also needed to address claims of how ensemble and summary displays are used beyond geospatial weather forecasting. Hurricanes are an example of geospatial data forecasting involving movement over space and time. It is possible that interpretations of ensemble versus summary displays differ across data dimensionality (e.g., 1-D bar charts or violin plots, see Correll & Gleicher, 2014) as well as across domains. For example, GPS-location data visualizations elicit top-down influences that can modify viewers’ judgments (Hegarty, Friedman, Boone, & Barrett, 2016). However, it is unclear if viewers of weather forecasting data visualizations demonstrate the same top-down influences. Additionally, the current studies provided limited information about the nature of the displays. This may have led viewers to rely more on visually salient features than they would have if provided with more specific instructions highlighting common misconceptions about uncertainty visualizations, including that changes in size of the display can represent other information than physical size changes and that ensemble members are not always an exhaustive representation of all of the data. If we had given participants more information about what the cone or ensemble represents, they might have misinterpreted it less. Future work could add supplemental instruction before display presentation and assess how effectively that information facilitates desired interpretations. Other biases may have resulted from the specific visual information depicted in the display. Perceptual biases and limitations of the visual system, such as simultaneous contrast effect and just noticeable differences, were not controlled for. Prior work shows that perception interacts with visualization techniques (e.g., Cleveland & McGill, 1986; Kosara & Skau, 2016). As such, future work is needed to generalize these findings beyond a geospatial context and to other visualization techniques.

## Conclusions

While there is disagreement about the optimal ways to visualize ensemble data, our work argues that both summary and ensemble displays have inherent biases based on their salient visual features. We propose that summary displays of geospatial uncertainty can be misinterpreted as displaying size information, while ensemble displays of the same information are not subject to this bias. On the other hand, when participants use ensemble displays to make point-based judgments, they may overweight individual ensemble members in their decision-making process. Overall, both user expertise and the intended visualization goal should be considered when visualization scientists decide to implement either summary or ensemble displays to communicate uncertainty. Current practice in visualization tends to emphasize the development of visualization methods more than testing usability (Isenberg, Isenberg, Chen, Sedlmair, & Möller, 2013), although there is a growing acknowledgment of the importance of incorporating human cognition and performance in visualization research (Carpendale, 2008; Kinkeldey, MacEachren, Riveiro, & Schiewe, 2015; Plaisant, 2004). As data availability and associated uncertainty visualization techniques continue to expand across the academic, industry, and public spheres, scientists must continue to advance the understanding of end-user interpretations in order for these visualizations to have their desired impact.

## Abbreviations

MLM: multilevel model
NHC: National Hurricane Center

## Footnotes

1 The odds for the *γ*_*00*_ intercept and subsequent odds ratio for the γ_10_ term are extreme values due to the far oil rig only being chosen in 12 out of 688 trials when the close rig was on a line.

2 The odds for the *γ*_*00*_ intercept and subsequent odds ratio for the γ_10_ term are extreme values due to the far oil rig only being chosen in 1 out of 481 trials when the close rig was on a line.
